# Systematic Identification of Target Genes for Cellular Morphology Engineering in *Synechococcus elongatus* PCC7942

**DOI:** 10.3389/fmicb.2020.01608

**Published:** 2020-07-09

**Authors:** Mingyi Zhang, Cuncun Qiao, Guodong Luan, Quan Luo, Xuefeng Lu

**Affiliations:** ^1^Key Laboratory of Biofuels, Qingdao Institute of Bioenergy and Bioprocess Technology, Chinese Academy of Sciences, Qingdao, China; ^2^Shandong Provincial Key Laboratory of Synthetic Biology, Qingdao Institute of Bioenergy and Bioprocess Technology, Chinese Academy of Sciences, Qingdao, China; ^3^University of Chinese Academy of Sciences, Beijing, China; ^4^Dalian National Laboratory for Clean Energy, Dalian, China; ^5^Laboratory for Marine Biology and Biotechnology, Qingdao National Laboratory for Marine Science and Technology, Qingdao, China

**Keywords:** cyanobacteria, cellular morphology, riboswitch, photosynthesis, cellular length

## Abstract

Cyanobacteria are serving as promising microbial platforms for development of photosynthetic cell factories. For enhancing the economic competitiveness of the photosynthetic biomanufacturing technology, comprehensive improvements on industrial properties of the cyanobacteria chassis cells and engineered strains are required. Cellular morphology engineering is an up-and-coming strategy for development of microbial cell factories fitting the requirements of industrial application. In this work, we performed systematic evaluation of potential genes for cyanobacterial cellular morphology engineering. Twelve candidate genes participating in cell morphogenesis of an important model cyanobacteria strain, *Synechococcus elongatus* PCC7942, were knocked out/down and overexpressed, respectively, and the influences on cell sizes and cell shapes were imaged and calculated. Targeting the selected genes with potentials for cellular morphology engineering, the controllable cell lengthening machinery was also explored based on the application of sRNA approaches. The findings in this work not only provided many new targets for cellular morphology engineering in cyanobacteria, but also helped to further understand the cell division process and cell elongation process of *Synechococcus elongatus* PCC7942.

## Introduction

Cyanobacteria emerged as the simplest and the most ancient oxygen-evolving phototrophs, paving the way for evolution of other aerobiont on the planet, and meantime contributing a large portion of the oxygen to the current biosphere environment (Flombaum et al., 2013; Rousseaux and Gregg, 2014). The flexible physiological and metabolic networks permit cyanobacteria significant potentials to acclimate to changeable environments and diverse ecosystems, including land, ocean, fresh water and polar regions (Waterbury et al., 1979; Demarsac and Houmard, 1993). By performing high efficient photosynthesis, cyanobacteria capture solar energy and carbon dioxide for production of diverse organic compounds, accounting for up to 20% of the primary production within the scope of the global (Hagemann, 2011; Flombaum et al., 2013; Rousseaux and Gregg, 2014). Not only that, cyanobacteria also play important and active roles in the global cycle of other important elements such as nitrogen, phosphorus, and iron (Sohm et al., 2011; Fernandez-Juarez et al., 2019; Wang et al., 2019).

In recent years, due to the unique characteristics such as efficient photosynthesis, rapid growth, simple structure, and convenient genetic manipulations, cyanobacteria serve as promising microbial platforms for artificially designing, constructing, and controlling photosynthesis-driven routes for directional conversion of energy and materials (Lu, 2010; Desai and Atsumi, 2013). Based on the development and utilization of system biology technologies (multiple -omics approaches), massive information about the metabolic profiles and dynamics of cyanobacteria cells under stable and changing environments have been acquired (Aikawa et al., 2019; Lin et al., 2019). Notable improvements in developing efficient tools of synthetic biology and metabolic engineering over the last decade have permitted effective regulation and expansion of the photosynthetic metabolism network (Sun et al., 2018b; Santos-Merino et al., 2019). Through assembling and regulating the native, heterologous, or artificial metabolic pathways in cyanobacteria chassis cells, photosynthetic production of dozens of natural or non-natural metabolites utilizing solar energy and carbon dioxide has been achieved with diverse cyanobacteria cell factories (Desai and Atsumi, 2013). Since far, some of the cyanobacteria cell factories products could be synthesized and accumulated at levels of g/L, accounting for up to 70% of the intracellular photosynthetic carbon flow (Gao et al., 2012, 2016a; Liu et al., 2019). Besides the synthesis capacity of final products, there are some other important traits of the cyanobacteria cell factories influencing the economic competitiveness of the photosynthetic biomanufacturing technology, including the tolerance to environmental stresses, the resistance to biocontaminants, and the convenience for biomass harvesting (Luan and Lu, 2018). To remove the restrictions over practical applications of photosynthetic biomanufacturing, these complex industrial traits of the cyanobacterial cell factories are yet to be significantly improved, which would require comprehensive remodeling of the behaviors and characteristics of the cyanobacteria chassis cells.

Cellular morphology is a basic and essential characteristic of cyanobacteria, as well as other microorganisms, significantly determining some of the important industrial properties of the derived cell factories. Previously it has been reported that modifications on cell surface facilitated cyanobacteria cells to survive in grazing of predators or infection of cyanophages (Xu et al., 1997; Simkovsky et al., 2012). In addition, the size and shape of cyanobacteria cells also significantly influenced the grazing resistances (Young, 2006; Jezberova and Komarkova, 2007) and the recovery characteristics of the photosynthetic cell factories, which is of great significance for economic feasibilities of industrially leveled photosynthetic biomanufacturing (Zamalloa et al., 2011; Chisti, 2013). Engineering cell sizes or shapes through manipulating the node genes influencing or determining cell morphogenesis provided a promising approach to optimize industrial properties of microbial cell factories (Jiang and Chen, 2016). In heterologous cell factories derived from *Escherichia coli* (*E. coli*) and *Halomonas campaniensis*, enlarged cell sizes and volumes significantly improved the yield of the PHB products and decreased the difficulties in biomass harvesting processes (Wang et al., 2014; Jiang et al., 2015, 2017). With *Synechococcus elongatus* PCC7942 (hereafter PCC7942 for short), a model strain of fresh water cyanobacteria, the concept of “morphology engineering” has also been confirmed. Through controllable expression of the components in Min system (which is participated in regulation of FtsZ protein and the Z-ring structure determining cell morphogenesis), cell lengths of PCC7942 could be extended from several micrometers to near millimeter levels. The elongated cells showed normal-gravity induced sedimentation behaviors and enhanced fragilities with mechanical treatments, which were both expected to enable convenient biomass harvesting and downstream processing in scaled cultivations (Jordan et al., 2017).

To facilitate more accurate and controllable editing of cellular morphology in cyanobacteria as required by an ideal robust industrial process, the identification of effective target genes is as important as the development of manipulation tools. In recent years, more and more accessible synthetic biology tools, including riboswitch (Ohbayashi et al., 2016), CRIPSRi (Yao et al., 2016), and microRNA tools (Sun et al., 2018a), have been developed and widely adopted in cyanobacteria engineering, permitting smart and rapid regulation of target genes. As compared, more effective node genes for morphology engineering in cyanobacteria are yet to be explored and evaluated. In this work, we performed systematic evaluation of the potential genes participating in cell morphogenesis of PCC7942. Combining knockout and overexpression manipulations, the influences of manipulating the corresponding genes on cell sizes and shapes were systematically evaluated and compared. The results provide useful information for designing cyanobacteria cell factories with smartly regulated morphology in future.

## Results and Discussion

### Screening the Potential Genes Involved in Cell Morphogenesis of PCC7942

As for rod-shaped bacteria, represented by *E. coli* and *Bacillus subtilis* (*B. subtilis*), cellular morphology is influenced simultaneously by the activities and process of cell division and elongation (Wang et al., 2014; Jiang and Chen, 2016; Jordan et al., 2017). Thus, the genes participating in the two machineries might contribute to the process and outcome of cell morphogenesis, while the disturbance of the expression pattern of these genes might cause significant changes in cell sizes and shapes. Among the morphogenesis machineries of rod-shaped bacterial cells, FtsZ and MreB serve as the most important cellular skeleton proteins, functioning in recruiting and orchestrating the subsequent components in the divisome and elongasome, respectively (Rohs et al., 2018). In PCC7942, two highly conserved homologous of *ftsZ* (*Synpcc7942_2378*) and *mreB* (*Synpcc7942_0300*) have been annotated on the chromosome, with amino acids sequence identities of as high as 49 and 56% to the homologous of *E. coli*, respectively (Table 1).

**TABLE 1:** Selection and engineering of candidate genes potentially participating in cell morphogenesis in *Synechococcus elongatus* PCC7942.

Proteins known to regulate cell shape			Homologous proteins in *Synechococcus elongatus* PCC7942									

Protein	Host strain	Identity (Query cover) with *S. elongatus*	Protein	Gene ID	Gene Knockout/Knockdown				Gene Overexpression			
					Cell length relative to WT (%)	Cell area relative to WT (%)	Cell shape	Growth relative to WT (% ± SD)	Cell length relative to WT (%)	Cell area relative to WT (%)	Cell shape	Growth relative to WT (% ± SD)
FtsZ	*E. coli*	49% (98%)	FtsZ	*Synpcc7942_2378*	Filamentous	Filamentous	Filamentous	55 ± 29	46	39	Short rod-shaped	48 ± 5
PPIA	*E. coli*	32% (77%)	Cdv1	*Synpcc7942_0653*	513	500	Elongated	96 ± 13	130	121	WT	85 ± 19
RodA	*E. coli*	33% (94%)	RodA	*Synpcc7942_1104*	53	102	Round	106 ± 6	130	118	WT	90 ± 20
MreB	*E. coli*	56% (96%)	MreB	*Synpcc7942_0300*	61	133	Round	83 ± 17	95	106	Spindle-shaped	70 ± 1
FtsE	*E. coli*	43% (97%)	FtsE	*Synpcc7942_1414*	95	99	WT	89 ± 3	133	118	WT	102 ± 7
SepF	*B. subtilis*	32% (90%)	Cdv2	*Synpcc7942_2059*	168	203	Elongated	98 ± 3	114	91	WT	92 ± 26
ZipN	*Synechocystis*	42% (72%)	ZipN	*Synpcc7942_1943*	Filamentous	Filamentous	Filamentous	98 ± 8	623	666	Elongated	104 ± 6
Cdv3	*Synechocystis*	35% (75%)	Cdv3	*Synpcc7942_2006*	542	732	Elongated	83 ± 2	576	760	Elongated	89 ± 3
SulA	*Synechocystis*	56% (99%)	SulA	*Synpcc7942_2477*	123	134	WT	95 ± 7	124	103	WT	98 ± 0
Ftn6	*Synechocystis*	37% (37%)	Ftn6	*Synpcc7942_1707*	855	1155	Filamentous	107 ± 2	141	115	WT	70 ± 2
FtsI	*Synechocystis*	46% (94%)	FtsI	*Synpcc7942_0482*	Filamentous	Filamentous	Filamentous	100 ± 9	130	128	WT	93 ± 12
FtsW	*Synechococcus sp.* PCC 7002	49% (93%)	FtsW	*Synpcc7942_0324*	Filamentous	Filamentous	Filamentous	29 ± 0	124	104	WT	68 ± 13

**E. coli*, *Escherichia coli*; *Synechocystis*, *Synechocystis* sp. PCC 6803; *B. subtilis*, *Bacillus subtilis*; Gene ID, Gene identifier in CyanoBase; Chl*a*, Chlorophyll *a* [Log_2_(Day 7 Chl*a*/Day 3 Chl*a*)];% identity (Query cover) with *S. elongatus* was calculated based on BLASTP program.*

To facilitate the cellular division process, FtsZ, a tubulin-like GTPase protein, would polymerize to form the Z-ring structure as the skeleton and scaffold of the cellular divisome complex. In cells of typical rod-shaped bacteria, including *E. coli* and *B. subtilis*, multiple components, including FtsA/ZipA/ZapB/SepF, FtsE, FtsK, FtsQ, FtsL, FtsB, FtsW, FtsI, and FtsN, would be further recruited to activate the cellular division process (Errington et al., 2003; Marbouty et al., 2009). FtsA, as an important and conserved cytoplasmic actin-like protein in multiple bacterial species, is responsible for stabilizing the Z-ring structure and recruiting subsequent proteins (Pichoff and Lutkenhaus, 2002). In cyanobacteria, FtsA is missing, while another protein termed as ZipN (*Synpcc7942_1943* in PCC7942), was discovered to work as an FtsA-like orchestrator for divisome assembly (Koksharova and Wolk, 2002; Marbouty et al., 2009; Camargo et al., 2019). A Cdv2 protein (*Synpcc7942_2059*), with 32% sequence similarity to SepF of *B. subtilis* (Table 1), has also been identified on the chromosome of PCC7942, which might bring additional contribution to the stabilization of the Z-ring structure (Miyagishima et al., 2005). Some other conserved cellular divisome components, including FtsE (*Synpcc7942_1414*), FtsW (*Synpcc7942_0324*), and FtsI (*Synpcc7942_0482*), have also been annotated in PCC7942, while the other portion are not detected in cyanobacteria (Miyagishima et al., 2005). Previously, it has been reported that the deficiency of these genes participating in cellular divisome led to filamentation of the mutant cells (Miyagishima et al., 2005). Some negative factors, including MinCDE, DivlVA, EzrA, SulA, and Noc would also participate in regulating the cell division process in rod-shaped bacteria by directly interacting with FtsZ to regulate or position the Z-ring structure (Goehring and Beckwith, 2005; Margolin, 2005). The MinCDE system (*Synpcc7942_2001*, *Synpcc7942_0220*, and *Synpcc7942_0897*), and the SulA protein (*Synpcc7942_2477*) has been identified on the chromosome of PCC7942 (Miyagishima et al., 2005; Koksharova and Babykin, 2011). And the Cdv3 protein (*Synpcc7942_2006*), which was reported to be involved in PCC7942 cell division, shows sequence similarity to the DivlVA from *B. subtilis* (Miyagishima et al., 2005). In addition, a periplasm located protein Cdv1 (*Synpcc7942_0653*), with high similarity to peptidyl-prolyl *cis*-*trans* isomerase (PPlase) was also recognized as a factor related with cell division, although the detailed mechanisms are yet elucidated (Miyagishima et al., 2005). Besides, there are also two cyanobacteria-specific proteins, Ftn6 (*Synpcc7942_1707* in PCC7942) with unknown functions and CikA as the circadian input kinase, identified to influence cell division process (Koksharova and Wolk, 2002; Miyagishima et al., 2005).

In addition to the factors participating in constructing and regulating the cell division process, the synthesis of peptidoglycan, the main component of cell wall, is also an important contributor to cell morphogenesis in *E. coli*. The non-canonical transglycosylase protein RodA has been reported to be involved in the regulation of cell shapes and lengths in many bacterial species, through interaction with the MreB skeleton (Henriques et al., 1998; Arora et al., 2018; Rohs et al., 2018). In PCC7942, the *Synpcc7942_1104* gene was annotated to encode the functional homolog of RodA.

**FIGURE 1:**
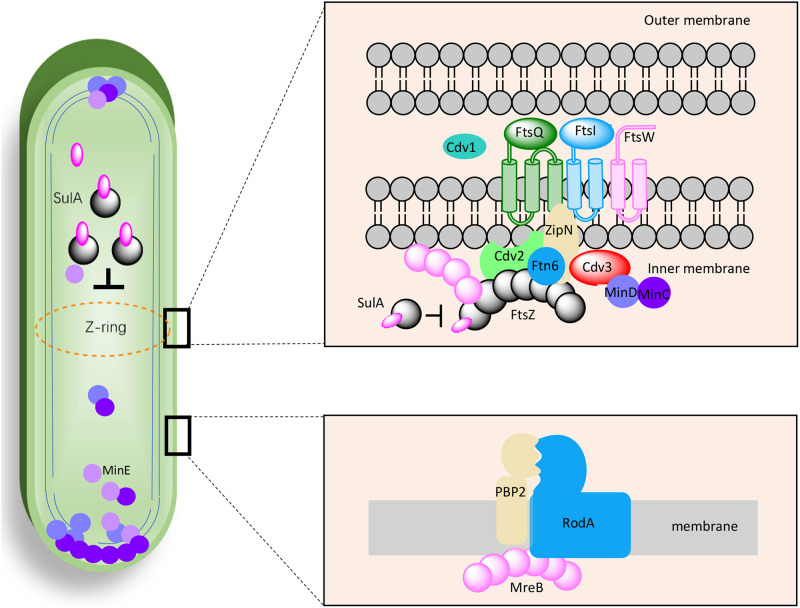
Proposed working model for the factors potentially participating in cell morphogenesis of PCC7942. The interactions, locations, and components were selected and illustrated referencing previous discoveries in *Escherichia coli*, *Bacillus subtilis*, and *Synechocystis* sp. PCC6803.

As mentioned above, the effects of genetic manipulations of MinCDE on cellular morphology in PCC7942 have been elucidated in a previous research (Jordan et al., 2017). Thus, in this work we main evaluated the effects of manipulating the other genes on cell morphogenesis engineering. The detailed information about the candidate genes have been summarized in Table 1 and the potential interrelationships among the corresponding proteins are presented in Figure 1 based on previous results and hypotheses.

### Exploring the Effects of Knocking Out/Down the Candidate Genes on Cell Morphogenesis of PCC7942

**FIGURE 2:**
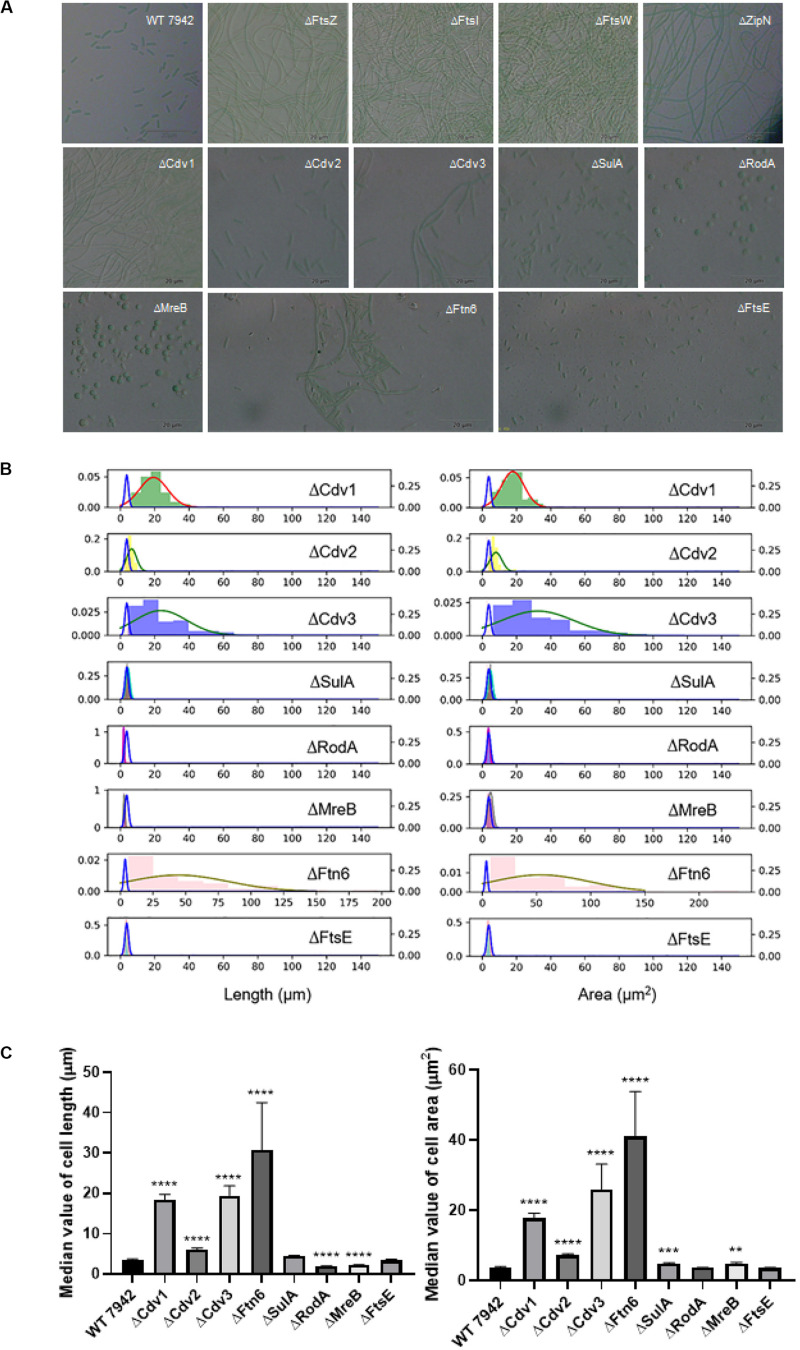
The disruption effects of the candidate genes on cellular morphology of PCC7942. **(A)** Microscopic photo of the wild type PCC7942 and disruption mutants. **(B)** The distribution histogram and normal distribution curve of the cell length (left) and the cell area (right) in the mutant strains compared with the wild-type strain (blue line). At least 100 cells were evaluated for statistical analysis. The left and right *Y* axes represent the distribution probability of the mutants and the wild type, respectively. **(C)** The comparison of cell length (left) and cell area (right) between wild type and mutant strains based on the value of median with 95% CI. The Kruskal-Wallis test in One-way ANOVA was used to analyze the significant difference. * indicates <0.05; ** indicates <0.01; *** indicates <0.001; **** indicates <0.0001.

To explore promising targets for morphology engineering, we first tried to knock out the twelve candidate genes (as listed in Figure 1 and Table 1) through homologous recombination in PCC7942 and to observe the changes of cellular morphology of the mutants with microscope. Due to the essential roles on cell division and cell elongation, the *ftsZ* and *mreB* genes could not be completely eliminated from the chromosome of PCC7942. Thus, homozygous transformants carrying complete disruption of *ftsZ* and *mreB* were not obtained through several attempts in this work. The result of quantitative PCR showed that the *ftsZ* and *mreB* mutants still remained the wild type genes (i.e., *ftsZ* and *mreB*) with ratios of about 76 and 39%, respectively (Supplementary Figures S1A, S2). The other ten mutants were successfully constructed as designed (Table 1 and Supplementary Figure S1A). In consistence with the crucial role as skeleton bricks of the cell divisome, the deficiency of FtsZ in PCC7942 caused significant influence on cellular morphology, resulting in filamentous cells (Table 1 and Figure 2A). Cells of the three other mutant strains deficient of subsequent cellular divisome components, including FtsI, FtsW, and ZipN, were also filamented, which could not be effectively calculated for cell lengths and areas under microscope (Figure 2A). In addition to the above four mutants with filamentous cells, the cell lengths and areas of the other eight mutants (ΔCdv1, ΔCdv2, ΔCdv3, ΔSulA, ΔRodA, ΔMreB, ΔFtn6, and ΔFtsE) were calculated and summarized in Table 1 and Figures 2B,C. The Cdv2 was predicated to perform similar functions of ZipN by stabilizing the structure of Z-ring, while when *cdv2* was disrupted, the effects on cellular morphology of PCC7942 was not as significant as that of *zipN*. The cell length and area of the ΔCdv2 mutant was just increased by 1.7- and 2-fold, respectively than these of the wild type (Table 1). Knockout of *ftsE*, another potential factor involved in cell divisome, caused minor changes in cell size and cell shape. The general phenomenon of cell filamentation in the deficient mutants of the components participating in the cellular divisome suggested the essential role of cell division on maintaining the short rod shape of PCC7942 cells, by preventing the formation of excessively long cells from the horizontal axis.

As compared, RodA and MreB displayed more significant importance on maintaining of cell shape from vertical axis. When RodA and MreB was eliminated, respectively, in PCC7942, the cell shapes of the disruption mutants were remodeled from short rod to sphere (Figure 2A), which might resulted from the impaired synthesis of cell wall and the loss of vertical tension. In addition, although cell length of the ΔMreB mutant was reduced by 40% comparing with that of the wild type, the cell area was not adversely affected, but enlarged by 1.3-fold (Table 1 and Figure 2B), which is similar with the phenotypes of *mreB* knocking-down mutants of *E. coli* (Kruse et al., 2003). The cell length of the ΔRodA mutant was reduced by 47% while the cell area of the mutant was still maintained on the same level of the wild type control (Figure 2C).

Moreover, the cells of PCC7942 mutants carrying ΔFtn6, ΔCdv3, and ΔCdv1 also showed significant elongation by 8. 6-, 5. 4-, and 5.1-fold of the wild type cell length, and the cell area were also enlarged by 11. 6-, 7. 3-, and 5.0-fold, respectively (Table 1), which is in consistence with previous reports (Miyagishima et al., 2005). A noteworthy point is that the elimination of Ftn6, Cdv3, and Cdv1 resulted in unequal division of the mutant cells (Figure 2A), suggesting that these proteins might contribute to the accurate positioning of Z-ring in cell membranes. The cell lengths and areas of the mutant cells deficient in SulA, another potential inhibitor of Z-ring structure, were just slightly influenced, both increased by 1.3-fold than these of the wild type cells (Table 1 and Figures 2B,C).

Deficiency or weakening of the genes participating in morphogenesis revealed different effects on cell growth of PCC7942. The ΔFtsZ and ΔFtsW mutants showed severely impaired growth, which decreased by 45 and 71% compared to that of the wild type strain (Table 1), indicating possible defection of cell divisions (Boyle et al., 1997; Sarcina and Mullineaux, 2000). In addition, the mutants carrying *mreB* and *cdv3* deficiencies also showed retarded growth rates of about 83% compared to that of wild type (Table 1), suggesting their potential roles in maintaining normal cell growth and division. As a key effector not only regulating shape determination but also patterning cell-wall growth, MreB is essential for cell viability, and *mreB* depletion resulted in loss of rod-shape and eventually cell lysis in *E. coli* (Gital et al., 2005; Kruse et al., 2005). Regarding Cdv3, previously it has been reported that partially knockout of this gene in *Synechocystis* reduced the growth rate by 50% (Marbouty et al., 2009). Other mutants did not show notable changes in cell growth and photosynthesis compared to the wild type PCC7942 (Table 1 and Supplementary Figure S3).

### Exploring the Effects of Overexpressing the Candidate Genes on PCC7942 Cell Morphogenesis

**FIGURE 3:**
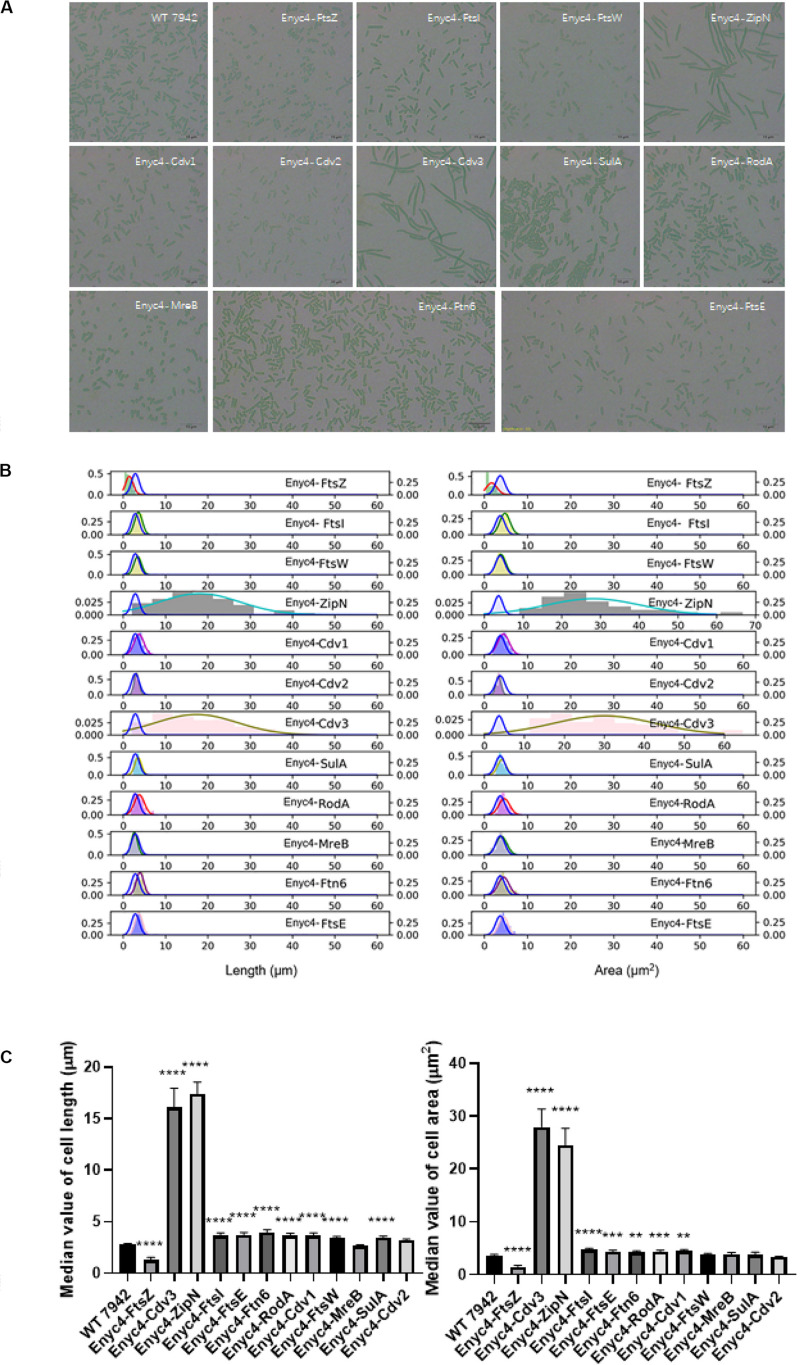
The overexpression effects of the candidate genes on cellular morphology of PCC7942. **(A)** Microscopic photo of the wild type PCC7942 and overexpression mutants. **(B)** The distribution histogram and normal distribution curve of the cell length (left) and the cell area (right) in the mutant strains compared with the wild type strain (blue line). At least 100 cells were evaluated for statistical analysis. The right left and right *Y* axes indicates the probability of the mutants and the wild type, respectively. **(C)** The comparison of cell length (left) and cell area (right) between wild type and riboswitch-regulated strains based on the values of median and the SEC. The Kruskal-Wallis test in One-way ANOVA was used to analyze the significant difference. * indicates <0.05; ** indicates <0.01; *** indicates <0.001; **** indicates <0.0001.

**FIGURE 4:**
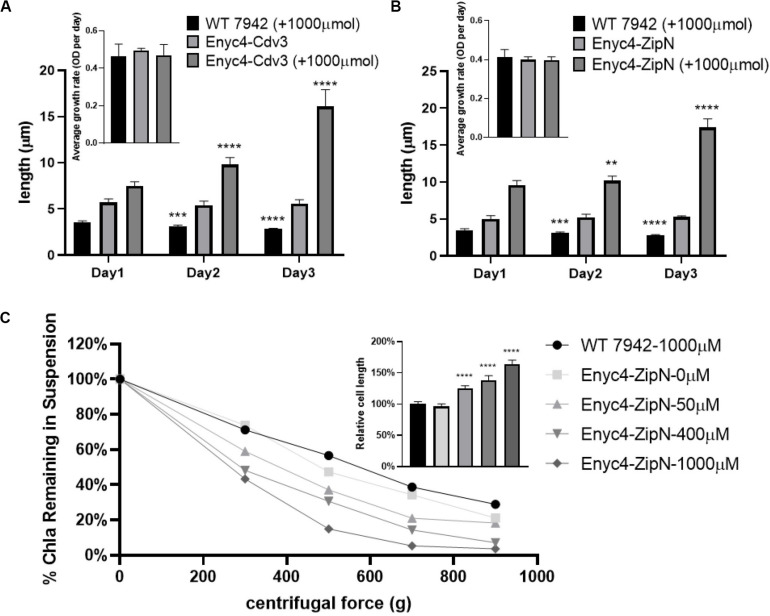
Response of the cell lengths to theophylline induction in PCC7942 strains carrying riboswitch-regulated expression of Cdv3 and ZipN. The cell lengths of the PCC7942 mutants carrying Ptrc-ENYC4-Cdv3 **(A)** and Ptrc-ENYC4-ZipN **(B)** were calculated during 3 days cultivation with (1000 μM) or without theophylline induction. The inset figures show the growth rates of the wild type and mutant strains during the process. The Kruskal-Wallis test in One-way ANOVA was used to analyze the significant difference of cell length at Day 2 (and Day 3) compared with that of at Day 1 in each strain. * indicates <0.05; ** indicates <0.01; *** indicates <0.001; **** indicates <0.0001. The culture of wild-type control was supplemented with 1000 μM theophylline, which didn’t affect its growth. **(C)** At Day 5 of induction by theophylline, Chl*a* (Chlorophyll *a*) contents in the supernatant of wild type and different Enyc4-ZipN cultures were analyzed after centrifugation. Ratio of Chl*a* content under the applied centrifugal force relative to the control condition (centrifugal force 0 g) was calculated for each culture. The embedded graph shows ratios of the cell lengths of the Enyc4-ZipN strain compared to the wild type strain.

In addition to the strategy of gene knockout, we also explored the effects of overexpressing each of the twelve candidate genes on cellular morphology of PCC7942. All the candidates were cloned and placed under control of a flexible gene expression system, consisting the *Ptrc*-promoter and a theophylline responsive riboswitch ENYC4 (Ohbayashi et al., 2016). The cassettes were subsequently integrated on the neutral site 2 (NS2) on the chromosome of PCC7942. Genotypes of the mutants were confirmed by PCR and DNA sequencing (Supplementary Figure S1A). As shown in Supplementary Figure S1B, cellular morphology of the PCC7942 wild type strain was not significantly influenced by the induction dose and induction time of theophylline, thus we calculated and compared the cell sizes and shapes of the overexpression strains after 3 days induction with 1 mM theophylline (Table 1 and Figure 3A). Overexpression of FtsZ resulted in the formation of significantly minimized cells, with 54% reduced cell lengths and 61% reduced cell area. This result is inconsistent with the phenomenon previously reported that constitutive expression of FtsZ (by *Ptrc-*promoter) in PCC7942 led to the generation of filamented cells (Mori and Johnson, 2001; Cohen et al., 2018). Previously similar counterintuitive phenomenon has been discovered in *E. coli* cells that 2–7-folds enforced expression of FtsZ resulted in minimized cells, while when the expression level was further enhanced to 12-fold higher, the cells would be remodeled into filamentous pattern (Ward and Lutkenhaus, 1985). A possible explanation could be that excessive FtsZ in appropriate range (with sufficient subsequent factors) accelerated the assembly of Z-ring and over-activated cell division, which in turn promoted the generation of minimized cells. While when the expression level of FtsZ was further increased, the abundances of the other cellular divisome components would become relatively insufficient. This might subsequently impair the formation and function of cell divisome and elongate the mutant cells. A similar mechanism could also explain the phenomenon that the overexpression of Cdv3 and ZipN resulted in significantly increased cell size (5.8- and 6.2-folds increased lengths than that of the wild type control, as shown in Table 1 and Figures 3B,C), because excessive abundances of specific factors for the cell division might competitively block and inhibit the interaction and affinity of the subsequent cells, subsequently impaired the normal cell division and generated elongated cells (Gao et al., 2017). An additional phenotype supporting this hypothesis is the increased frequency of uneven cell divisions (Figure 3A). The response mode of cellular morphology to theophylline induction in these two mutants were further explored. As shown in Figures 4A,B, when no theophylline was added, cell lengths of the mutants carrying additional copy of *zipN* or *cdv3* (NS2-Ptrc-ENYC4-ZipN and NS2-Ptrc-ENYC4-Cdc3) were maintained well (1.6- and 1.8-fold higher than that of the wild type), while when 1 mM theophylline was added, cell lengths of the mutant cells kept increasing to about 4-fold of the wild type level. The addition of theophylline and the elongation of the cells did not caused significant influence on cell growth (Figures 4A,B inset charts). In addition, not only on the time scale, the cell length also exhibited a good response to the concentration gradient of theophylline (Figure 4C). Taking Enyc4-ZipN as an example, when the theophylline concentration was gradually increased, the cell length and the cell sedimentation rate were both synchronously increased, indicating the potential of this strategy for application in controllable biomass harvesting (Jordan et al., 2017).

Comparing with the FtsZ-overexpression strain, the strain overexpressing MreB, another important cell morphogenesis skeleton bricks, showed little difference in cell lengths and areas from that of the wild type control, except that the MreB-overexpression cells tended to be spindle (Figure 3A). Excessive accumulation of other factors potentially involved in cell morphogenesis, including Cdv1, RodA, FtsE, FtsI, FtsW, SulA, and Ftn6, caused similar effects on cell length, with increase ranging from 1.2- to 1.4-fold, while rod-shapes of the cells were maintained (Table 1 and Figure 3A). Although it has been reported previously that overexpression of SepF in *B. subtilis* resulted in filamentation of the mutant cells and eventually cell deaths (Gao et al., 2017), the excessive abundance of the homologous protein Cdv2 in PCC7942 caused minor effects on cellular morphology (with 10% increased cell lengths and 10% decreased cell area, Table 1). This indicated that the Cdv2/SepF was not as essential as other components such as ZipN and Cdv3 for Z-ring stability and functionality in PCC7942.

Some of the overexpression mutants exhibited growth impairment phenotypes similar to the knockout mutants. For example, the growths rates of the strains overexpressing FtsZ, MreB, FtsW, and Ftn6, were significantly reduced by 52, 30, 32, and 30%, respectively, compared to the wild type control (Table 1). As mentioned above, the mismatch in concentrations and timing of different morphogenesis components might result in disorder and disturbance of the Z-ring assemble and functionalities. The subsequent impairments on formation and function of cell divisome might further cause the weakening of cell survival and proliferations (Chiu et al., 2008). It is noteworthy that at least for a portion of the potential genes participating in morphogenesis (FtsW, MreB, and FtsW), artificial (either up- or down-) regulation of the abundances resulted in significantly weakened cell growth (Table 1), and these effects should not be resulted from morphology changes, because growths of both the elongated (ΔFtsZ) and the shortened (ENYC4-FtsZ) were similarly reduced. The detailed mechanisms are yet to be disclosed, while possible influence on cyanobacteria cultivation process should be taken into consideration when the strategy of morphogenesis engineering is adopted.

### Adopting Small RNA (sRNA) Based Gene Repression Approach to Regulate Cellular Morphology of PCC7942

**FIGURE 5:**
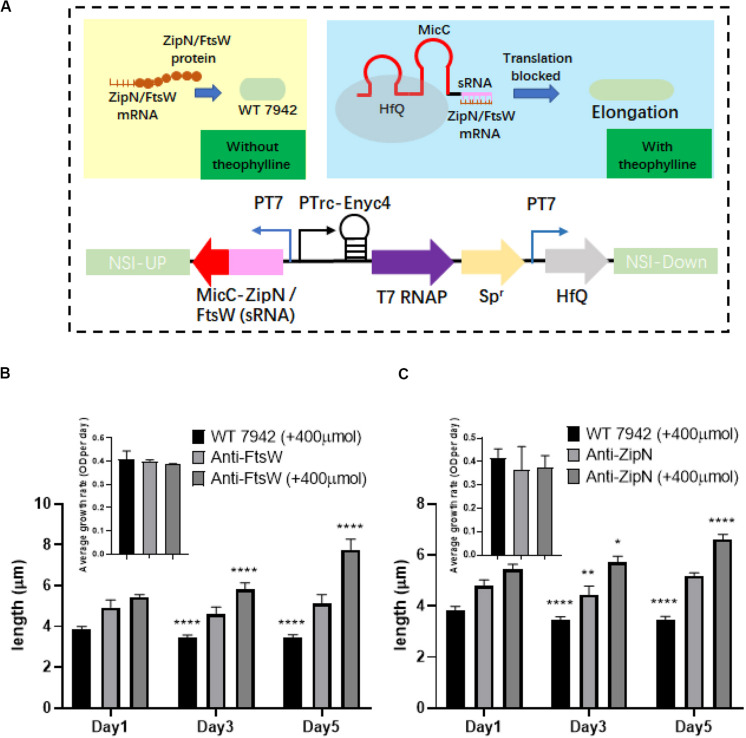
Regulation of PCC7942 cell lengths through the adoption of sRNA based gene repression approach. **(A)** Design of the Hfq-MicC sRNA approach for controllable repression of ZipN or FtsW to regulate cell lengths of PCC7942. **(B)** The dynamics of cell lengths of the PCC7942-Anti-FtsW **(B)** and PCC7942-Anti-ZipN **(C)** strains induced with 400 μM theophylline for 3 days. The inset figures represent the growths of the wild type control and the mutant strains. The Kruskal-Wallis test in One-way ANOVA was used to analyze the significant difference cell length at Day 2 (and Day 3) compared with that at Day 1 for each strain. * indicates <0.05; ** indicates <0.01; *** indicates <0.001; **** indicates <0.0001.

By adopting the theophylline-responsive riboswitch approach, we achieved flexible regulation of cell length of PCC7942 by inducible overexpression of important contributors involved in cell division. Subsequently, we also attempted to regulate cellular morphology of PCC7942 through controllable down-regulation of potential targets. sRNA regulatory tools are promising metabolic engineering approaches to repress the expression of both endogenous and exogenous target genes (Nakashima et al., 2006). In recent years, this approach has also been successfully developed and adopted in cyanobacteria for remodeling cellular metabolism (Li et al., 2018; Sun et al., 2018a). In this work, we aimed to adopt Hfq-MicC tool (Sun et al., 2018a) to regulate the expression of ZipN and FtsW. The components and working mechanism of the controllable gene repression system is illustrated in Figure 5A, in which sRNA-MicC cassette and the Hfq protein were placed under the control of the T7 promoter, while the heterologous T7 RNA polymerase was driven by the previously utilized theophylline-responsive expression system (*Ptrc*-ENYC4). When the expression of T7 RNA polymerase was induced by addition of theophylline, the sRNA-MicC RNA would be subsequently transcribed and bind to the mRNA of the target gene, resulting in controllable gene silencing. Targeting ZipN and FtsW, two mutant strains were further constructed (termed as Anti-ZipN and Anti-FtsW, respectively). As shown in Figure 5, when 400 μM theophylline was added into the medium, cell lengths of the Anti-ZipN and Anti-FtsW strains were significantly increased by 1.3- and 1.5-fold than theoe of the cells without theophylline induction, respectively (Figures 5B,C). However, there are still two obvious drawbacks of this system. When no theophylline was supplemented, leak expression of T7 RNA polymerase or Hfq-MicC system might partially inhibit the expression of ZipN and FtsW, leading to slightly increased cell lengths of the mutant strains (by 1.3- and 1.4-fold higher than the wild type level). While when theophylline was added, the elongations of the mutant cells was not as significant as that in the disruption mutants (filamentous cells of the *ΔZipN* and *Δ*FtsW mutants). In future, the development of more stringent and smart gene repression approach enabling a wider regulatory space could be expected to bring in more desirable control system for cellular morphology.

## Conclusion

Cyanobacteria are promising microbial chassis for photosynthetic biomanufacturing in future, and optimization of industrial properties of cyanobacteria chassis cells and engineered strains are necessary for developing economically competitive photosynthetic cell factories (Gao et al., 2016b; Luan and Lu, 2018). Cellular morphology engineering is an up-and-coming strategy to improve complex phenotypes required by industrial application. In this work, we performed systematic exploration of promising target genes for engineering cellular morphology of an important cyanobacteria strain, *Synechococcus elongatus* PCC7942. Previously, the knockout strategy was adopted to identify the influence of target genes on cell morphogenesis, while the overexpression strategy was relatively rarely utilized. Aiming to get a more clear and comprehensive map of potential nodes in cellular morphology engineering, we combined the knockout/down and overexpression strategies targeting each of the twelve potential genes participating in cell division and/or elongation. The influence of elevated and decreased abundance of the targets on cell morphogenesis were systematically calculated and compared, illustrating a more clear and comprehensive map of cellular morphology engineering nodes. As the most important skeleton bricks for Z-ring structure, the expression level of FtsZ show strongly negatively regulatory effects on cell length of PCC7942. The cells of the FtsZ defective mutant were filamented and the overexpression of FtsZ resulted in generation of minimized cells. However, the disruption and overexpression of the other components involved in cell division both elongated cells of the respective mutants, which might be resulted from imbalance of the ratios among diverse components. As compared, MreB and RodA, the two factors contributing to the cell wall synthesis, show more significant influence on cell shape of PCC7942. Shapes of the two disruption mutants (MreB and RodA defective strains) were remodeled from rod into sphere. In addition, the overexpression of MreB also led to the formation of spindle-shaped cells. Adopting a previously developed sRNA based expression regulation approach, we partially achieved flexible and controllable regulation of the PCC7942 cell lengths, and more desirable regulatory effects could be expected through development and application of more power synthetic biology toolbox.

## Materials and Methods

### Chemicals and Reagents

Chemicals utilized in this work were purchased from Sinopharm Chemical Reagent Co., Ltd. (Shanghai, China) except theophylline from Sigma-Aldrich (St. Louis, MO, United States). *Taq* and *FastPfu* Fly DNA polymerases for PCR and pEASY-Blunt Cloning kits were obtained from Transgene Biotech (Beijing, China). Restriction enzymes and T4 DNA ligase were purchased from Thermo Fisher (Waltham, MA, United States). Oligonucleotides synthesis and DNA sequencing was processed by TsingKe (Qingdao, China).

### Strain Construction and Cultivation

*Escherichia coli* DH5α was used as the host strain for plasmids construction and grown in LB media at 37°C. The wild-type strain of PCC7942 is a gift of Prof. Xudong Xu from Institute of Hydrobiology, Chinese Academy of Sciences. To construct the knockout plasmids, the respective upstream and downstream homologous fragments of each target gene were amplified by PCR, and subsequently fused with *aacC1* (Gentamicin-resistance gene, GmR, 1.2 kb) by fusion PCR. The fused fragment was then cloned into pEASY-blunt simple vector. The generated plasmids were transformed into the PCC7942 wild type cells. Gentamicin resistant transformants were obtained after 7 days cultivation on selective BG11 agar plates (containing 10 μg/ml gentamicin). Genotypes of the transformants were confirmed by PCR and DNA sequencing. To overexpress target genes, the backbone of plasmid was amplified from previously developed plasmid (Qiao et al., 2018), containing upstream and downstream homologous fragments of NS2 (neutral site 2 on the chromosome of PCC7942), the *Ptrc-*ENYC4 promoter, chloramphenicol resistant gene (CmR, 0.95 kb). Then the PCR amplicons of the target genes were digested with restriction enzyme (*Pac*I/*Pae*I) and ligated into the backbone plasmid. Chloramphenicol-containing BG11 agar plates were used to select resistant transformants. The plasmid used for synthetic sRNA expression was obtained from Prof. Weiwen Zhang of Tianjin University. Synthetic sRNAs that recognize specific sequences of target genes were introduced into plasmid through site-directed mutagenesis as previously reported (Yoo et al., 2013). And here spectinomycin resistance was used as the phenotypes to isolate positive transformants. All information about strains, plasmids and oligonucleotides was presented in Supplementary Table S1. All of the PCC7942 derived strains were grown in BG11 medium in flasks that were incubated on a horizontal rotary shaker at 150 rpm at 30°C under constant white-light illumination of 30 μmol/m^2^/s. Theophylline was added to liquid media when necessary.

### Microscopy and Cell Length Measurements

All images were captured using Olympus BX51 microscope (100X/1.3 Oil Ph3) with an Olypus DP72 camera. For cell length measurements, three independent biology parallels with at least 250 cells per image were recorded and further measured by manual tools in DP2-BSW3 software (Olympus, Japan).

### Growth, Chlorophyll *a* (Chl*a*) and Oxygen Evolution Measurements

Growths rates (shown in Table 1) were calculated based on equation Log [(Day 7 Chl*a*/Day 3 Chl*a*), 2], while growths in Figures 4, 5 were shown by OD_730_ per day. In growth measurement for gene overexpression strains, 1 mM theophylline was added. Chl*a* was extracted by cell suspension in equal volume methyl alcohol overnight at 4°C. Then Chl*a* content was determined by equation 12.9447^∗^(A665-A720) with methyl alcohol as control. Oxygen evolution was measured under light intensity of 143 μmol/m^2^/s using a Clark-type oxygen electrode (Hansatech, British) connected to the Oxy Lab software, final data were divided by Chl*a* content of each culture.

## Data Availability Statement

All datasets generated for this study are included in the article/Supplementary Material.

## Author Contributions

MZ performed the experiments and participated in manuscript preparation. CQ and QL participated in the research. QL, GL, and XL designed and directed the research. GL and XL wrote and revised the manuscript. All authors contributed to the article and approved the submitted version.

## Conflict of Interest

The authors declare that the research was conducted in the absence of any commercial or financial relationships that could be construed as a potential conflict of interest.
