# Supplementary material for: Systematic Identification of Target Genes for Cellular Morphology Engineering in Synechococcus elongatus PCC7942
Source: Front Microbiol. 2020 Jul 9;11:1608. doi: 10.3389/fmicb.2020.01608 (PMC7381316; doi:10.3389/fmicb.2020.01608)
Supplement: Supplementary file 1 [file Table_1.DOCX]

Supplementary Table S1. The oligonucleotides used in this study.

| Primers | Sequences (5’-3’) |
| --- | --- |
| Ftsz-up-1 | ACCATGATTACGCCAAGCTGCCCTTCTCCAAACCAACCCTCGCTA |
| Ftsz-up-2 | TGTCCCCTTATACACAAGGAAGGCGGTCATTAGGAATAGTG |
| Ftsz-GmR-1 | ACTATTCCTAATGACCGCCTTCCTTGTGTATAAGGGGACAC |
| Ftsz-GmR-2 | GCTCAGATATGGCGTGGAGTGGCCGGGAAGCCGATCTCG |
| Ftsz-down-1 | CCGAGATCGGCTTCCCGGCCACTCCACGCCATATCTGAGC |
| Ftsz-down-2 | TATAGGGCGAATTGAAGCTGCCCTTAACCACTTCCATAATTTCGGC |
| FtsI-up-1 | ACCATGATTACGCCAAGCTGCCCTTagcaggtcattgaaccacgg |
| FtsI-up-2 | TGTCCCCTTATACACAAGGAgaaatagcgattggcgtcgt |
| FtsI-GmR-1 | acgacgccaatcgctatttcTCCTTGTGTATAAGGGGACAC |
| FtsI-GmR-2 | cgaccccggctcatagagatGGCCGGGAAGCCGATCTCG |
| FtsI-down-1 | CCGAGATCGGCTTCCCGGCCatctctatgagccggggtcg |
| FtsI-down-2 | TATAGGGCGAATTGAAGCTGCCCTTCTTCCGCCATTTGCTTGACA |
| FtsW-up-1 | ACCATGATTACGCCAAGCTGCCCTTCAGCCCTTTCCCTAGAACGA |
| FtsW-up-2 | TGTCCCCTTATACACAAGGAtgagaatgatgccgagggtc |
| FtsW-GmR-1 | gaccctcggcatcattctcaTCCTTGTGTATAAGGGGACAC |
| FtsW-GmR-2 | ccgccaatcactgtcaacagGGCCGGGAAGCCGATCTCG |
| FtsW-down-1 | CCGAGATCGGCTTCCCGGCCctgttgacagtgattggcgg |
| FtsW-down-2 | TATAGGGCGAATTGAAGCTGCCCTTACAGAACAGACCGGATTGGC |
| ZipN-up-1 | ACCATGATTACGCCAAGCTGCCCTTTGCGTATTCCTCTCGATTAC |
| ZipN-up-2 | TGTCCCCTTATACACAAGGACTTCCGATTCAAACAGCAAC |
| ZipN-GmR-1 | GTTGCTGTTTGAATCGGAAGTCCTTGTGTATAAGGGGACAC |
| ZipN-GmR-2 | GTTTCCTCATCTTGGCTCTGGGCCGGGAAGCCGATCTCG |
| ZipN-down-1 | CCGAGATCGGCTTCCCGGCCCAGAGCCAAGATGAGGAAAC |
| ZipN-down-2 | TATAGGGCGAATTGAAGCTGCCCTTTAGACCTGCTCAACTTCATC |
| Cdv1-up-1 | ACCATGATTACGCCAAGCTGCCCTTCAGCAACTATTTTTGGGCGATC |
| Cdv1-up-2 | TGTCCCCTTATACACAAGGACTTGAACCACAAAGGGCTG |
| Cdv1-GmR-1 | GCAGCCCTTTGTGGTTCAAGTCCTTGTGTATAAGGGGACAC |
| Cdv1-GmR-2 | AAGGGTAAGTAGCGTGGCTGGGCCGGGAAGCCGATCTCG |
| Cdv1-down-1 | CCGAGATCGGCTTCCCGGCCCAGCCACGCTACTTACCCTT |
| Cdv1-down-2 | TATAGGGCGAATTGAAGCTGCCCTTCCGCCTTGCTCACGAAAATG |
| Cdv2-up-1 | ACCATGATTACGCCAAGCTGCCCTTAGCTTTGCCTCCAGGTGAAG |
| Cdv2-up-2 | TGTCCCCTTATACACAAGGAGGGTTCCATTACCACCACTTC |
| Cdv2-GmR-1 | AAGTGGTGGTAATGGAACCCTCCTTGTGTATAAGGGGACAC |
| Cdv2-GmR-2 | GCTCCATCATCGTCAGGTTCGGCCGGGAAGCCGATCTCG |
| Cdv2-down-1 | CCGAGATCGGCTTCCCGGCCGAACCTGACGATGATGGAGC |
| Cdv2-down-2 | TATAGGGCGAATTGAAGCTGCCCTTAGCTGATCCGCCGTTACCTG |
| Cdv3-up-1 | ACCATGATTACGCCAAGCTGCCCTTTTGAACGCCATCTATCCTG |
| Cdv3-up-2 | TGTCCCCTTATACACAAGGATACTGTTTTGGTCAATCAGC |
| Cdv3-GmR-1 | GCTGATTGACCAAAACAGTATCCTTGTGTATAAGGGGACAC |
| Cdv3-GmR-2 | CATTCCTGTTGAAGGGTTTGGGCCGGGAAGCCGATCTCG |
| Cdv3-down-1 | CCGAGATCGGCTTCCCGGCCCAAACCCTTCAACAGGAATG |
| Cdv3-down-2 | TATAGGGCGAATTGAAGCTGCCCTTCTCGACATCAAAAACTTGCC |
| SulA-up-1 | ACCATGATTACGCCAAGCTGCCCTTACTGATGACCGACGTACTGC |
| SulA-up-2 | TGTCCCCTTATACACAAGGAcccactcactgttaccgctt |
| SulA-GmR-1 | aagcggtaacagtgagtgggTCCTTGTGTATAAGGGGACAC |
| SulA-GmR-2 | ttccaattcgcagggtcaccGGCCGGGAAGCCGATCTCG |
| SulA-down-1 | CCGAGATCGGCTTCCCGGCCggtgaccctgcgaattgga |
| SulA-down-2 | TATAGGGCGAATTGAAGCTGCCCTTCCGGTCTCTGTTTCGGTAGG |
| RodA-up-1 | ACCATGATTACGCCAAGCTGCCCTTGACATTTTTGGCTCTGGCGG |
| RodA-up-2 | TGTCCCCTTATACACAAGGAgggcgagagcatcagcaata |
| RodA-GmR-1 | tattgctgatgctctcgccc[RodA-up]TCCTTGTGTATAAGGGGACAC |
| RodA-GmR-2 | gccacgcAaaactcttccagGGCCGGGAAGCCGATCTCG |
| RodA-down-1 | CCGAGATCGGCTTCCCGGCCctggaagagtttTgcgtggc |
| RodA-down-2 | TATAGGGCGAATTGAAGCTGCCCTTAATGCTGCCAGTTGAGGGAG |
| MreB-up-1 | ACCATGATTACGCCAAGCTGCCCTTATCAGGCTCATCATTGCGGC |
| MreB-up-2 | TGTCCCCTTATACACAAGGAATCATCGTCCCTGTCGGCT |
| MreB-GmR-1 | GAGCCGACAGGGACGATGATTCCTTGTGTATAAGGGGACAC |
| MreB-GmR-2 | GACTTTCTTGAGGTACTGCGGGCCGGGAAGCCGATCTCG |
| MreB-down-1 | CCGAGATCGGCTTCCCGGCCCGCAGTACCTCAAGAAAGTCC |
| MreB-down-2 | TATAGGGCGAATTGAAGCTGCCCTTTGCCACCAGCTATCGACGCT |
| Ftn6-up-1 | ACCATGATTACGCCAAGCTGCCCTTGGATTACGCATCGTTCCC |
| Ftn6-up-2 | TGTCCCCTTATACACAAGGAGTAACGGCCTTGGTAAATC |
| Ftn6-GmR-1 | CGATTTACCAAGGCCGTTACTCCTTGTGTATAAGGGGACAC |
| Ftn6-GmR-2 | TGCCACAGAGCAAGGATTTGGGCCGGGAAGCCGATCTCG |
| Ftn6-down-1 | CCGAGATCGGCTTCCCGGCCCAAATCCTTGCTCTGTGGC |
| Ftn6-down-2 | TATAGGGCGAATTGAAGCTGCCCTTGGATTAGGAGAAATGGCTTGA |
| FtsE-up-1 | ACCATGATTACGCCAAGCTGCCCTTTGGCAAGCTCTACCTGTTCTG |
| FtsE-up-2 | TGTCCCCTTATACACAAGGAagcatcacattctcgaccgc |
| FtsE-GmR-1 | gcggtcgagaatgtgatgctTCCTTGTGTATAAGGGGACAC |
| FtsE-GmR-2 | ccgctcctgctgagaaatgcGGCCGGGAAGCCGATCTCG |
| FtsE-down-1 | CCGAGATCGGCTTCCCGGCCgcatttctcagcaggagcgg |
| FtsE-down-2 | TATAGGGCGAATTGAAGCTGCCCTTGCTACCGGGCAATGTTCTGC |
| ENYC4-FtsZ-f | CCTTAATTAAATGACCGACCCTATGCCGAT |
| ENYC4-FtsZ-r | ATTTGCATGCCTAGGGTCGGTTTTGAATTTTCCG |
| ENYC4-FtsI-f | CCTTAATTAAatgacggtactgccgcaagc |
| ENYC4-FtsI-r | ATTTGCATGCtcaattctggttaggagcca |
| ENYC4-FtsW-f | CCTTAATTAAatgagtcttgccctccccc |
| ENYC4-FtsW-r | ATTTGCATGCCGATTTTATCGAGGTctaggcc |
| ENYC4-ZipN-f | CCTTAATTAAGTGCGTATTCCTCTCGATTACTACC |
| ENYC4-ZipN-r | ATTTGCATGCCTAGCGCACCAAACTAATCG |
| ENYC4-Cdv1-f | CCTTAATTAAATGCGATCGCTGATCCAAGTTG |
| ENYC4-Cdv1-r | ATTTGCATGCTCATGGGCGTTTGAGGTTGT |
| ENYC4-Cdv2-f | CCTTAATTAAGTGTCTTTTGTGAACCGGAT |
| ENYC4-Cdv2-r | ATTTGCATGCCTATTGAGCAGCGTAGCGGC |
| ENYC4-Cdv3-f | CCTTAATTAAGTGACCCAAGCCCAATCACT |
| ENYC4-Cdv3-r | ATTTGCATGCTTAGCGCGATCGCCGACG |
| ENYC4-SulA-f | CCTTAATTAAgtgaagattgcgattacggg |
| ENYC4-SulA-r | ATTTGCATGCctagggcaggaattgctgc |
| ENYC4-RodA-f | CCTTAATTAAatgctggctcgtggcttgcg |
| ENYC4-RodA-r | ATTTGCATGCtcagtagcggctgatctgcc |
| ENYC4-MreB-f | CCTTAATTAAGTGAGTATTCTTCGGCGCTTC |
| ENYC4-MreB-r | ATTTGCATGCCTAGGCGCGGGAGCTGGCAC |
| ENYC4-Ftn6-f | CCTTAATTAAGTGACCTCTACCCGTACTGC |
| ENYC4-Ftn6-r | ATTTGCATGCCTACCCAGCGCAATCGCTGC |
| ENYC4-FtsE-f | CCTTAATTAAatggcagaatccgcgcctcc |
| ENYC4-FtsE-r | ATTTGCATGCtcaaaaggtagctgcaaggac |
| ZipN-f | AGTGCGTATTCCTCTCGATTACTACCCTATAGTGAGTCGTATTAggatcc |
| ZipN-r | GTAGTAATCGAGAGGAATACGCACTTTCTGTTGGGCCATTGCATTGC |
| FtsW-f | AatgagtcttgccctcccccaGtggCCTATAGTGAGTCGTATTAggatcc |
| FtsW-r | ccaCtgggggagggcaagactcatTTTCTGTTGGGCCATTGCATTGC |
| rnpB-f | AGCAAGGTGGAGGGACAA |
| rnpB-r | CGAAGACAGAGGGCAGTTAT |
| mreB-f | GCGGCACCACGGAAGTT |
| mreB-r | ATCGCCTCACTCAGCTCATC |
| gmR-f | CGCAGTGGCGGTTTTCATGG |
| gmR-r | ACATCGACCCACGGCGTAAC |
| ftsZ-f | AAGAAGTGGGTGCGCTGACG |
| ftsZ-r | TGCGGCTGTTCCTTCTTCCG |

Table S2. The strains used in this study.

| Strains | Genotype | Source |
| --- | --- | --- |
| PCC7942 | Wildtype | Institute of Hydrobiology CAS |
| ∆FtsZ | *∆ftsZ::GmR* | This Study |
| ∆FtsI | *∆ftsI:: GmR* | This Study |
| ∆FtsW | *∆ftsW:: GmR* | This Study |
| ∆ZipN | *∆zipN:: GmR* | This Study |
| ∆Cdv1 | *∆cdv1:: GmR* | This Study |
| ∆Cdv2 | *∆cdv2:: GmR* | This Study |
| ∆Cdv3 | *∆cdv3:: GmR* | This Study |
| ∆SulA | *∆sulA:: GmR* | This Study |
| ∆RodA | *∆rodA:: GmR* | This Study |
| ∆MreB | *∆mreB:: GmR* | This Study |
| ∆Ftn6 | *∆ftn6:: GmR* | This Study |
| ∆FtsE | *∆ftsE:: GmR* | This Study |
| Enyc4-FtsZ | *∆NSII::*P*_trc-Enyc4_*-*ftsZ-CmR* | This Study |
| Enyc4-FtsI | *∆NSII::*P*_trc-Enyc4_*-*ftsI-CmR* | This Study |
| Enyc4-FtsW | *∆NSII::*P*_trc-Enyc4_*-*ftsW- CmR* | This Study |
| Enyc4-ZipN | *∆NSII::*P*_trc-Enyc4_*-*zipN- CmR* | This Study |
| Enyc4-Cdv1 | *∆NSII::*P*_trc-Enyc4_*-*cdv1- CmR ^R^* | This Study |
| Enyc4-Cdv2 | *∆NSII::*P*_trc-Enyc4_*-*cdv2- CmR* | This Study |
| Enyc4-Cdv3 | *∆NSII::*P*_trc-Enyc4_*-*cdv3- CmR* | This Study |
| Enyc4-SulA | *∆NSII::*P*_trc-Enyc4_*-*sulA- CmR* | This Study |
| Enyc4-RodA | *∆NSII::*P*_trc-Enyc4_*-*rodA- CmR* | This Study |
| Enyc4-MreB | *∆NSII::*P*_trc-Enyc4_*-*mreB- CmR* | This Study |
| Enyc4-Ftn6 | *∆NSII::*P*_trc-Enyc4_*-*ftn6- CmR* | This Study |
| Enyc4-FtsE | *∆NSII::*P*_trc-Enyc4_*-*ftsE- CmR* | This Study |
| Anti-FtsW | *∆NSI::*P*_trc-Enyc4_*-*T7rnap-*T_r_*_bcl_-*P*_T7_-asftsw-micC-*P*_trc-Enyc4_-hfq-T_rbcl_-sp^R^* | This Study |
| Anti-ZipN | *∆NSI::*P*_trc-Enyc4_*-*T7rnap-*T_r_*_bcl_-*P*_T7_-aszipN-micC-*P*_trc-Enyc4_-hfq-T_rbcl_-sp^R^* | This Study |

*GmR*, gentamicin-resistant; C*mR*, chloramphenicol-resistant.

Figure S1


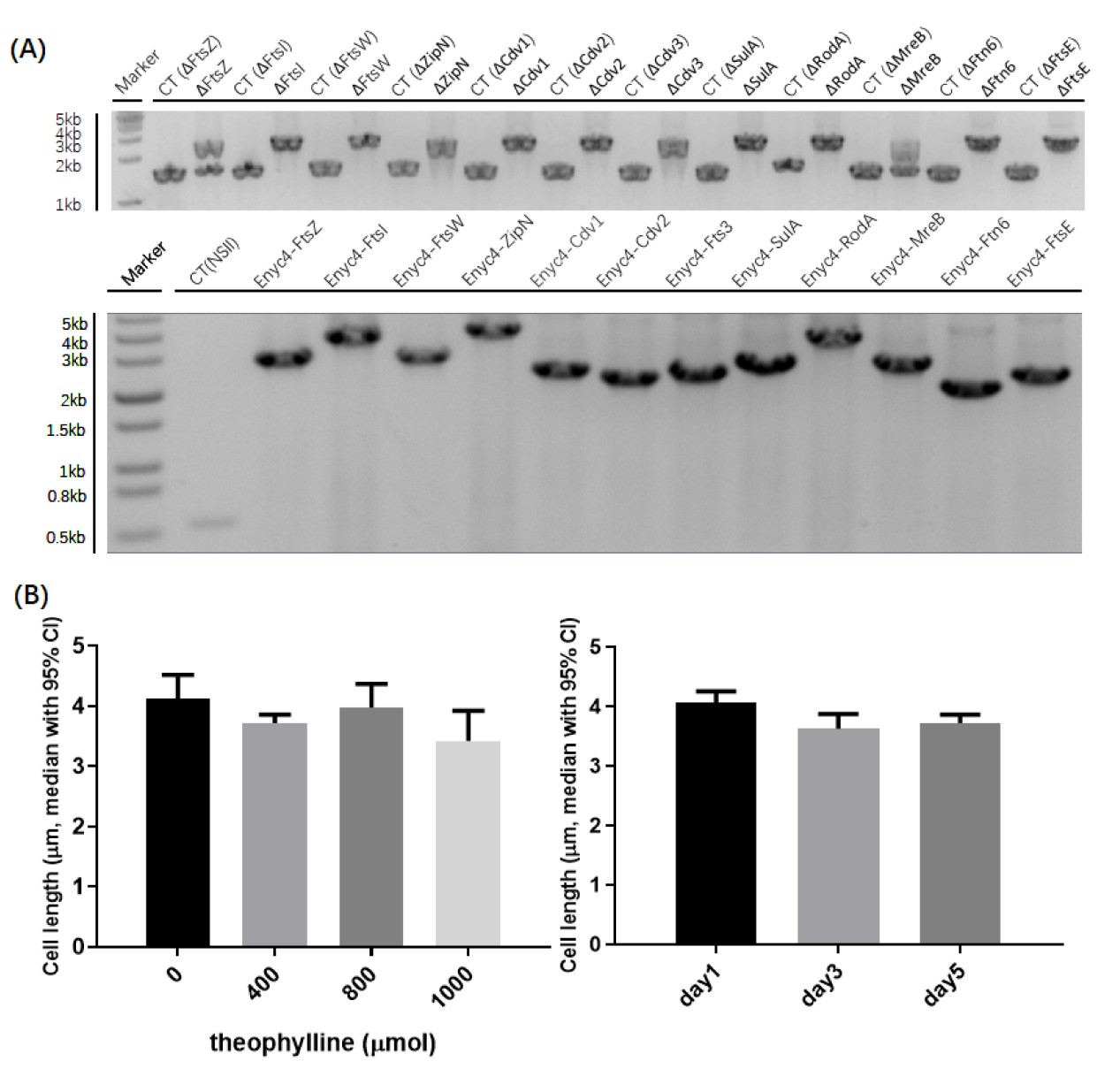


Figure S1. (A) Confirmation of the genotypes of the PCC7942 mutants carrying deficient or overexpressed candidate genes. (B) The left graph represents cellular lengths (micron, μm) response of PCC7942 wild type strain toward addition of gradient theophylline. The working concentration of theophylline in the right graph was 400 μmol, and columns represent cell length of PCC7942 wild type strain at different times. The initial OD_730_ for two graphs were both 0.5.

Figure S2


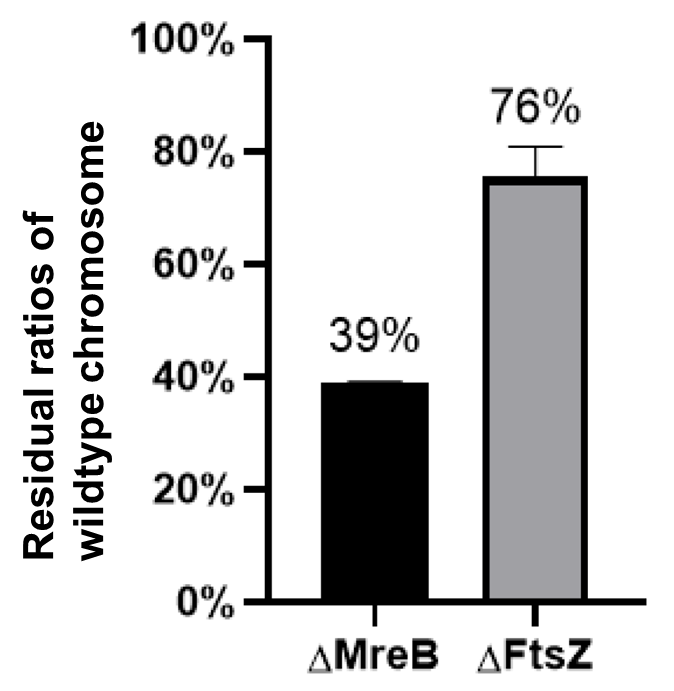


Figure S2. Determination of the results of disrupting mreB and ftsZ in PCC7942 by quantitative PCR (qPCR). Total DNA of the mutant and wild type strains of PCC7942 were extracted by Ezup bacterial genome DNA extraction kit (Sangon Biotech, Shanghai) for qPCR experiments. The reference gene was *rnpB* (105 bp). *mreB* fragment (91 bp) was amplified by primers (mreB-f/r) from the ∆MreB and wild type strains, while G*mR* (97 bp) and *ftsZ* fragmens (95 bp) were amplified, respectively, from the ∆FtsZ and wildtype strains with the corresponding primers (GmR-f/r, ftsZ-f/r). TB Green Premix Ex Taq II (Tli RNase H Plus, Takara) reagent was used for qPCR. Data represent the means ± standard deviations from two independent experiments.

Figure S3


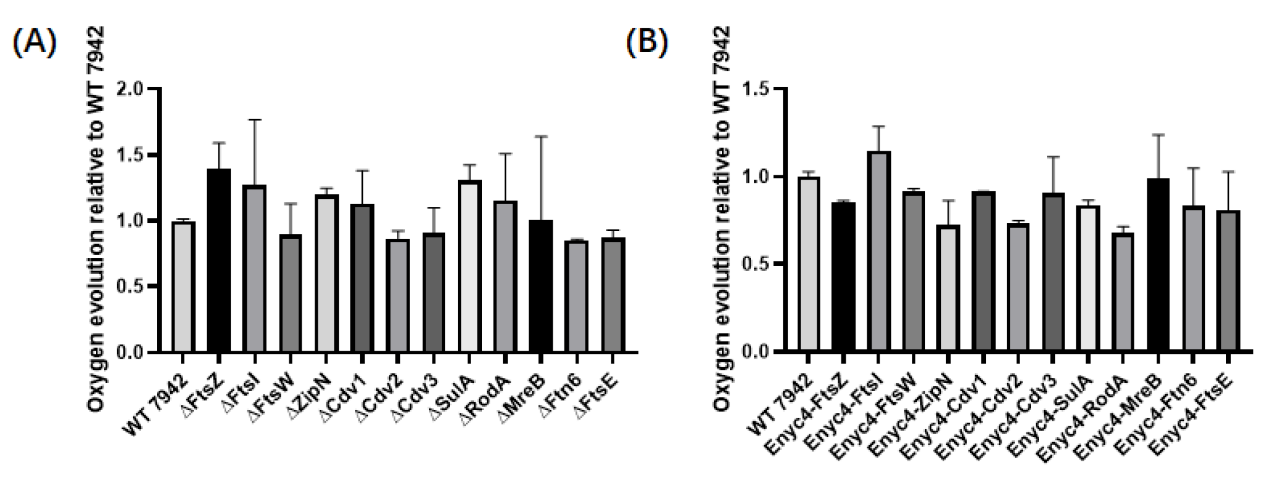


Figure S3. Comparison of the oxygen evolution rates of the knockout/down strains (A) or the overexpression strains (B) to the wild type PCC7942. Cyanobacterial cultures were sampled at Day3 after inoculation with initial OD_730_ 0.05. 1mM theophylline was added in the medium at inoculation (B).
